# Loneliness in Relation to Social Factors and Self-Reported Health Among Older Adults: A Cross-Sectional Study

**DOI:** 10.1177/21501319231198644

**Published:** 2023-09-12

**Authors:** Anna Axén, Elin Taube, Johan Sanmartin Berglund, Lisa Skär

**Affiliations:** 1 Blekinge Institute of Technology, Karlskrona, Sweden; 2 Malmö University, Malmö, Sweden

**Keywords:** community health, primary care, health promotion, lifestyle change, prevention

## Abstract

**Background:**

Loneliness is described as a public health problem and can be both a consequence of aging and a cause of ill health. Lonely older adults tend to have difficulties making new social connections, essential in reducing loneliness. Loneliness often varies over time, but established loneliness tends to persist. Maintaining good health is fundamental throughout the life course. Social connections change with aging, which can contribute to loneliness.

**Aim:**

This study aimed to investigate loneliness in relation to social factors and self-reported health among older adults.

**Method:**

A cross-sectional research design was used based on data from the Swedish National Study on Aging and Care, Blekinge (SNAC-B), from February 2019 to April 2021. Statistical analysis consisted of descriptive and inferential analysis.

**Results:**

Of n = 394 participants, 31.7% (n = 125) stated loneliness. Close emotional connections were necessary for less loneliness. Loneliness was more common among those who did not live with their spouse or partner and met more rarely. Furthermore, seeing grandchildren and neighbors less often increased loneliness, and a more extensive social network decreased loneliness.

**Conclusion:**

This study underlined the importance of social connections and having someone to share a close, emotional connection with to reduce loneliness.

## Introduction

Loneliness is a distinct experience defined as the state between the desired, expected degree of social connections and the actual degree. The experience differs between each person and situation depending on the stage in life and the societies in which they live.^1^ A well-established definition of loneliness is Weiss’s description of emotional and social loneliness as the absence of a valuable, emotional relationship with another person that can provide a sense of belonging.^2^ Existential loneliness is an experience of being fundamentally separated from others and the world.^3^ Loneliness can also be seen as an experience as a whole, beyond classification. It is well known that older adults are particularly prone to experience loneliness. A systematic review and meta-analysis indicate that nearly a third (28.5%) of those aged >60 years are lonely.^4^ The oldest old (>80 years) are almost twice as likely to be lonely compared to middle-aged working adults,^5^ which underlines the fundamental importance of social connections.^6^ Solid social connections are correlated to lower loneliness^7^ and a higher perceived purpose in life.^8^ Older adults experiencing loneliness tend to have more difficulty making new friends and consider fewer people to be friends. Thus, emotions and networks correlate.^6^ Loneliness varies over time, but perceived loneliness often persists.^2,6,9^ Loneliness can associate with a change in one’s relationship with the world, with others, through social ties, or with oneself.^10^ A solid social network with sufficient connections increases the likelihood of survival by 50% compared to older adults with few social connections and both subjective and objective aspects of social connections are essential.^11^ Loneliness can also be a consequence of aging, such as declining health,^12,13^ illness, and the possible loss of a life partner.^13^ Experiencing loneliness also increases the risk of rating one’s health lower.^14-16^ Feelings of belonging to others positively impact health.^17^ Remaining in the home is important, since the home symbolizes safety, security, or familiarity—this is expressed as *aging in place.*^18^ Aging is associated with factors such as retirement, possible relocation, and loss of family and friends. Aging impacts maintenance of health^19^; the consequences of aging may include reduced vision and hearing, making it socially challenging to form new connections and maintain existing relationships.^20,21^ Losing friends and family due to death or illness cuts connections and are seen as part of aging.^22^

Interventions for loneliness are ongoing in society, but research is needed on the impact of social relations on loneliness.^23^ This study contributes to the body of knowledge on the impact of loneliness on health in the older adults. Effective strategies for understanding and preventing loneliness need to be developed, and increased knowledge of loneliness, and related social factors, such as the size of the social network, the frequency and the quality of social connections, and self-reported health, can facilitate this process.

## Aim

The aim was to investigate loneliness in relation to social factors and self-reported health among older adults.

## Method

### Design

A cross-sectional research design was conducted based on data from the Swedish National Study on Aging and Care (SNAC), a longitudinal population-based multicenter study ongoing in 4 different geographical areas (Skåne, Blekinge, Kungsholmen, and Nordanstig) since 2001. SNAC includes participants aged ≥60 years and aims to increase knowledge about how environment and lifestyle affect aging and to focus on future care needs. A new cohort of 60-year-olds is added to the study population every 6 years. Participants are assessed every sixth year, except for the participants ≥78 years, who are assessed every third year.^24^

## Sample and Setting

A convenience sample (n = 394) was drawn from study participants examined from February 2019 to April 2021 in SNAC-B and represented urban and rural areas in a southeastern county in Sweden with approximately 65 000 inhabitants. The sample consisted of age clusters at 66, 72, 78, 84, 87, 90, 93, 96, and 99 years, all of which were included in the 18-year follow-up survey in SNAC-B (Table 1). The age clusters were divided into 2 different groups as loneliness may increase with age.^25^ Participants who completed a question about perceived loneliness and living in private housing (community dwelling) were selected for inclusion.

**Table 1.** Loneliness Related to Socio-demographics.

Variable (%)	Total sample n = 394	Lonely n = 125	Not lonely n = 269	*P*-value
Age-group				.659^b^
66, 72, and 78	294	91 (31.0)	203 (69.0)	
84, 87, 90, 93, 96, and 99	100	34 (34.0)	66 (66.0)	
Gender				.536^b^
Men	188	63 (33.5)	125 (66.5)	
Women	206	62 (30.1)	144 (69.9)	
Civil status*				.465^a^
Married	256	79 (30.9)	177 (69.1)	
Widowed/-er	72	25 (34.7)	47 (65.3)	
Unmarried	36	14 (38.9)	22 (61.1)	
Divorced	24	5 (20.8)	19 (79.2)	
Living arrangements*				.795^c^
Alone	128	42 (32.8)	86 (67.2)	
Together with spouse	257	81 (31.5)	176 (68.5)	
Other	2	1 (50.0)	1 (50.0)	

a Pearson’s chi-square test.

b Chi-square with continuity correction.

c Fisher’s exact test.

* Missing 0.8% to 4.1%.

## Measures

Both single items and validated instruments collected through questionnaires and structured interviews as part of a larger dataset were assessed, including socio-demographics (age, gender, civil status, and living arrangement; Table 1), loneliness, social factors, and self-reported health. The question measuring loneliness was, “*Do you ever feel lonely?*” *(no never, no rarely, yes sometimes* and *yes often).* The responses were dichotomized (*not lonely* and *lonely*). *Not lonely* was the response item *no never* or *no rarely* and *lonely* was *yes sometimes* or *yes often.* Single items measured the size of the social network, the frequency, and the quality of social connections. The prevalence of cardiovascular disease, cancer, and diabetes was measured by the self-reported health of the sample, which also measured the prevalence of medications, including non-prescribed and herbal medications. Health-related quality of life (HRQoL) was measured with EQ5D-3L, the EuroQol instrument,^26^ which consists of 5 different domains; *mobility, self-care, usual activities, pain and discomfort*, and *anxiety and depression (no problems, moderate problems, and extreme problems).* The EQ5D-3L questionnaire results in a 5-digit health summary that illustrates each dimension’s reported health problem, and the numbers are converted into an index value describing the health state. Health status is defined according to the population’s preferences in each country.^27^ The United Kingdom (UK) tariff time trade-off technique (TTO) was assessed,^26^ as no Swedish version exists. Converted responses have a utility score from 0.00 (worst health) to 1.00 (perfect health). Values below 0 occur and represent worse than death. Through use of a visual analog scale (VAS), the current self-reported health is scored on a scale of 0 to 100, where 0 means the worst possible condition and 100 the best possible conditions.^27^ The mini-mental state examination (MMSE), which ranks cognitive function on a scale of 0 to 30, was used; higher scores indicate better cognitive function.^28^ MMSE scores of 0 to 22 are signs of dementia, 23 to 26 are mild cognitive impairment, and 27 to 30 are normal cognitive functions.^29^

## Statistical Analysis

The descriptive analysis presented an overview of the distribution of the selected variables compared to stated loneliness. The inferential analysis included Pearson’s chi-square tests to compare responses to the dependent variable, loneliness (0/1), with variables on the nominal, ordinal, and interval-level scales. Fisher’s exact test was applied when the data contained values <5. Pearson’s chi-square with continuity correction was applied for 2 × 2 crosstabulations. The Mann-Whitney *U*-test was used to compare differences between 2 independent groups when the variable was ordinal but not normally distributed. Normal distribution was controlled using histograms. The dependent variable, loneliness, was dichotomized 0 = not lonely and 1 = lonely, a prerequisite for logistic regression.^30^ The value for rejecting the null hypothesis was set at *P* ≤ .05. All statistical analyses were conducted in SPSS (ver.28).

## Results

The sample had a mean age of 76.5 years (SD 7.1). The range 66 to 99 was divided into age group 1 (66, 72, 78), n = 294 (74.6%), and age group 2 (84, 87, 90, 93, 96, 99), n = 100 (25.4%); men n = 188 (47.7%), and women n = 206 (52.3%). The majority were married and lived with a spouse (Table 1).

Of the total sample of 394 participants, 125 (31.7%) stated that they felt loneliness *sometimes* or *often* (95% Cl 26.9%-36.3%), while 269 (68.3%) stated feeling loneliness *never* or *rarely*. There was a statistically significant difference in loneliness and the number of people the participants knew well and could talk to (*P* ≤ .001). The majority of those who stated loneliness knew 1 to 3 people (49.6%), followed by 4 to 6 people (36.0%) and those who knew >7 people (10.4%; Table 2). The connections when the participant lived separately from their spouse or partner differed between the lonely and not lonely participants. Some indicated never meeting their spouse or partner (17.0%), but most visited their spouse or partner daily (75.5%; *P* = .002). Most had contact with their grandchildren monthly or more rarely, indicating that loneliness is more prominent in those who interact less often with their grandchildren (*P* = .011). Furthermore, the lonely participants’ connections with neighbors were less frequent (*P* = .009; Table 2). Loneliness related to the quality of social connections showed a significant difference (*P* = .007) between the lonely and not lonely participants regarding those who had someone they felt they could be themselves in front of and who accepted them fully, with all their merits and shortcomings. Additionally, the majority reported having at least 1 person who could give them proper personal support to cope with life’s stresses and problems, but a few stated no or that they probably did not have such a person in their lives (*P* = .006). This illustrates that the lonely participant had fewer social connections of quality. The majority of the lonely participants felt a strong connection with their family (89.0%; *P* = .014; Table 2).

**Table 2.** Loneliness Related to the Size, Frequency, and Quality of Social Connections.

Variable^*^	Lonely n (%)	Not lonely n (%)	*P*-value
How many people do you think you know well and can talk to about things?			
0	5 (4.0)	3 (1.1)	**<.001** ^b^
1-3	62 (49.6)	91 (33.8)	
4-6	45 (36.0)	96 (35.7)	
>7	13 (10.4)	79 (29.4)	
How often do you see your. . . spouse/partner in person (not living together)			.**002**^b^
Every day	40 (75.5)	156 (90.2)	
Weekly	3 (5.7)	8 (4.6)	
Monthly or more rarely	(0.0)	3 (1.7)	
Never	9 (17.0)	4 (2.3)	
Don’t know	1 (1.9)	2 (1.2)	
How often do you see your. . . Grandchildren in person			.**011**^b^
Every day	(0.0)	7 (3.0)	
Weekly	30 (29.4)	101 (42.8)	
Monthly or more rarely	72 (70.6)	124 (52.5)	
Never	(0.0)	2 (0.8)	
Don’t know	(0.0)	2 (0.8)	
How often do you see your. . . Neighbor in person			.**009**^b^
Every day	33 (30.8)	91 (36.3)	
Weekly	34 (31.8)	108 (43.0)	
Monthly or more rarely	34 (31.8)	38 (15.1)	
Never	3 (2.8)	8 (3.2)	
Don’t know	3 (2.8)	6 (2.4)	
Do you have someone you feel you can be yourself in front of who accepts you with all your merits and shortcomings?			.**007**^b^
Yes. definitely	80 (64.0)	209 (77.7)	
Yes. probably	39 (31.2)	55 (20.4)	
No. probably not	4 (3.2)	5 (1.9)	
No. not at all	2 (1.6)	0 (0.0)	
Do you feel that you have someone or some people who can give you the proper personal support to cope with life’s stresses and problems?			.**006**^b^
Yes. definitely	65 (52.0)	171 (63.6)	
Yes. probably	51 (40.8)	94 (34.9)	
No. probably not	7 (5.6)	2 (0.7)	
No. not at all	2 (1.6)	2 (0.7)	
Do you feel a strong connection with your family (in addition to your spouse, partner, and children)?			
Yes. to a high or moderate degree	105 (89.0)	202 (77.7)	.**014**^a^
No. not particularly or not at all	13 (11.0)	58 (22.3)	

Significant value is highlighted in bold.

a Pearson’s chi-square test.

b Fisher’s exact test.

* Missing 0.5% to 50.8%.

In the reported conditions, a statistical significance was shown in the prevalence of transient ischemic attack (TIA; *P* = .012) and diabetes type 2 (*P* = .021), indicating a higher prevalence of these conditions in the lonely participants. However, these conditions were rare in the sample. Prescribed medicines were more prevalent in the lonely participants (*P* = .025; Table 3). A single EQ5D summary utility score with a cut-of-point of the mean (1.533, ranging from −0.132 to 2.0) was assessed to divide low and high HRQoL. Loneliness was not significantly related to low HRQoL, but lonely participants tended to rate their HRQoL as lower. There were no significant differences between the groups in the 5 dimensions of the EQ5D-3L. Of the 5 dimensions, Cronbach’s alpha was .62, ranging from .51 to .62. The mean MMSE score was lower in those who stated loneliness; however, this was not a significant difference (*P* = .114; Table 4). In age group 2, HRQoL was lower in both those who reported loneliness (65.5%) and those who did not (68.3%). Lonely men tended to rate their HRQoL as high (64.4%), while lonely women tended to rate their HRQoL as low (59.3%). Living alone and being lonely were related to low HRQoL (64.9%). Living with a spouse or partner and being lonely indicated a high HRQoL (59.5%), as did being married (58.4%) and unmarried (63.6%). Being a widow/widower was related to loneliness and lower HRQoL (70.8%; Table 5).

**Table 3.** Loneliness Related to Reported Conditions and Medications.

Variable	Total sample n = 394	Lonely n (%)	Not lonely n (%)	*P*-value
Reported conditions*				
Hypertension (current)	53 (13.8)	18 (15.0)	35 (13.3)	.765^b^
TIA (transient ischemic attack)	10 (2.6)	7 (5.8)	3 (1.1)	.**012**^a^
Diabetes type 2	15 (3.9)	9 (7.5)	6 (2.3)	.**021**^a^
Angina	11 (2.9)	2 (1.7)	9 (3.4)	.514^a^
Myocardial infarction	13 (3.4)	7 (5.8)	6 (2.3)	.123^b^
Cancer	37 (9.6)	8 (6.7)	29 (11.0)	.253^b^
Medications				
Prescribed*	339 (88.3)	113 (94.2)	226 (85.6)	.**025**^b^
Non-prescribed**	74 (19.4)	22 (18.3)	52 (19.8)	.835^b^
Herbal**	91 (23.8)	31 (25.8)	60 (22.9)	.620^b^

a Fisher’s exact test.

b Chi-square with continuity correction.

* Missing 2.5%.

** Missing 3.0%.

Significant values are highlighted in bold.

**Table 4.** Loneliness Related to HRQoL and MMSE.

Variable*	Total sample n = 394	Lonely n (%)	Not Lonely n (%)	*P*-value
EQ5D-3L n (%)				
High HRQoL	219 (57.8)	62 (52.5)	157 (60.2)	.202^b^
Low HRQoL	160 (42.2)	56 (47.5)	104 (39.8)	
EQ5D-3L VAS mean (SD)	72.8 (21.3)	72.9 (20.8)	72.7 (21.6)	.926^a^
EQ5D-3L n (%)				
Mobility				
No problem	273 (71.8)	82 (68.9)	191 (73.2)	.240^c^
Moderate problem	106 (27.9)	36 (30.3)	70 (26.8)	
Extreme problem	1 (0.3)	1 (0.8)	0 (0.0)	
Self-care				
No problem	375 (98.2)	116 (96.7)	259 (98.9)	.223^c^
Moderate problem	4 (1.0)	2 (1.7)	2 (0.8)	
Extreme problem	3 (0.8)	2 (1.7)	1 (0.4)	
Usual activities				
No problem	354 (92.6)	108 (90.8)	246 (93.9)	.435^c^
Moderate problem	23 (6.0)	9 (7.6)	14 (5.3)	
Extreme problem	4 (1.0)	2 (1.7)	2 (0.8)	
Pain and discomfort				
No problem	112 (29.3)	34 (28.3)	78 (29.8)	.154^a^
Moderate problem	248 (64.9)	75 (62.5)	173 (66.0)	
Extreme problem	22 (5.8)	11 (9.2)	11 (4.2)	
Anxiety and depression				
No problem	273 (71.5)	80 (66.7)	193 (73.7)	.279^c^
Moderate problem	103 (27.0)	37 (30.8)	66 (25.2)	
Extreme problem	6 (1.6)	3 (2.5)	3 (1.1)	
MMSE mean (SD)	28.1 (2.0)	27.9 (2.2)	28.3 (2.0)	.114^a^

a Mann-Whitney *U*-test.

b Chi-square with continuity correction.

c Fisher’s exact test.

* Missing 3.0 to 11.9.

**Table 5.** Loneliness Related to Socio-Demographic and HRQoL.

Variable*	High HRQoL	Low HRQoL	*P*-value
Age (years)			
(1) 66, 72, and 78			
Lonely	52 (58.4)	37 (41.6)	.100^a^
Not lonely	137 (69.2)	61 (30.8)	
(2) 84, 87, 90, 93, 96, and 99			
Lonely	10 (34.5)	19 (65.5)	.983^a^
Not lonely	20 (31.7)	43 (68.3)	
Gender			
Men			
Lonely	38 (64.4)	21 (35.6)	.807^a^
Not lonely	81 (67.5)	39 (32.5)	
Women			
Lonely	24 (40.7)	35 (59.3)	.088^a^
Not lonely	76 (53.9)	65 (46.1)	
Living arrangement			
Alone			
Lonely	13 (35.1)	24 (64.9)	.073^a^
Not lonely	45 (54.8)	38 (45.2)	
With spouse/partner			
Lonely	47 (59.5)	32 (40.5)	.614^a^
Not lonely	109 (63.7)	62 (36.3)	
Other			
Lonely	1 (100.0)	0 (0.0)	1.00^a^
Not lonely	0 (0.0)	1 (100.0)	
Civil status			
Married			
Lonely	45 (58.4)	32 (41.6)	.310^a^
Not lonely	113 (66.1)	58 (33.9)	
Unmarried			
Lonely	7 (63.6)	4 (36.4)	.538^a^
Not lonely	10 (45.5)	12 (54.5)	
Widower/widow			
Lonely	7 (29.2)	17 (70.8)	.185^a^
Not lonely	22 (48.9)	23 (51.1)	
Divorced			
Lonely	2 (50.0)	2 (50.0)	1.00^a^
Not lonely	10 (52.6)	9 (47.4)	

a Chi-square with continuity correction.

* Missing 3.8% to 5.3%.

The significant factor that emerged from the logistic regression analysis was the odds of stating loneliness as it increased by 3% for every year of age (*P* = .045). Furthermore, the odds of loneliness decreased as the size of the social network increased (*P* ≤ .001). Prescribed medications indicated increased odds of loneliness (*P* = .018; Table 6).

**Table 6.** Logistic Regression of Loneliness.

	OR	Lower 95% CI	Upper 95% CI	*P*-value
Size of social network and medications^a^				
How many people do you think you know well and can talk to about things?*	0.523	0.392	0.698	**<0.001**
Prescribed**	2.792	1.192	6.539	**0.018**
Demographic^b^				
Age (years)	1.030	1.001	1.061	**0.045**
Gender***	1.150	0.750	1.763	0.521

Abbreviations: CI, confidence interval; OR, odds ratio.

Dependent variable loneliness 0 = no; 1 = yes. Significant values are highlighted in bold.

* 1 = None; 2 = 1 to 3; 3 = 4 to 6; 4 = >7.

** 0 = No; 1 = Yes.

*** 0 = woman; 1 = men.

a Nagelkerke *R*^2^ = .097, Hosmer and Lemeshow test sig. = .686.

b Nagelkerke *R*^2^ = .016, Hosmer and Lemeshow test sig. = .107.

## Discussion

This present study indicated that 31.7% of older adults stated loneliness and that the risk of loneliness increases with age. The older age group tended to rate their self-reported health as low, whether they felt lonely or not, and loneliness was more prominent in the older age group. These results underline the importance of close connections for less loneliness do to the fact that previous research shows that loneliness increases with age.^25^ Not living with one’s spouse or partner increases loneliness, but seeing one’s grandchildren and neighbors more frequently may reduce it. The size of the social network is a significant positive factor in reducing loneliness. Additionally, people outside the immediate family are essential in reducing loneliness. Other research implies that loneliness is a risk factor for developing dementia.^31^ Research suggests that living with a spouse relates to a significantly lower risk of loneliness.^25^ Living alone, being a widow/widower, and being lonely were related to a low HRQoL.

Within the social network, relationships with children, grandchildren, and friends are seen as essential and provide meaningful feelings.^32,33^ Furthermore, ages ≥81 years tend to feel lonelier than ages 65 to 70 years,^25^ and the prevalence of loneliness and poor self-rated health increases with age.^34^ This study suggests that seeing grandchildren more frequently reduces loneliness; other research shows that looking after one’s grandchildren makes one feel less lonely.^35^ A high perceived quality of close connection decreases the risk of loneliness.^36^ Research implies that having a few extra friends reduces loneliness^6,37^ by around 10% for the average person.^6^ Thus, the focus should be on increasing the social network and the personal experience of social connections.^38^ Still, instead of focusing on the number of social connections, improving the quality of the present connections should be encouraged.^39^ Additionally, having few friends is associated with becoming lonelier over time,^6^ and the loss of social activities increases the risk of loneliness more than does a small network or poor health.^40^

Previous research has shown that less frequent social contact increases loneliness.^34^ This is in line with the findings of this study, which show that a more extensive social network and close connections with others can reduce loneliness. Research has found that the loneliest are those who live alone and report good health, while the lowest levels of loneliness were found in those who live with someone and report good health. Living alone with poor health is associated with 10 times higher odds of feeling lonely than living with someone and having good health.^25^ Initially, that loneliness and living alone are related to poorer subjective health.^41^ A higher degree of loneliness is related to lower HRQoL.^42^

Data were collected before and during the COVID-19 pandemic, and reported loneliness may have been affected by the shutdown, as society changed and social connections became limited. Other research implies that the social network decreased by 5% among older adults during the pandemic, forcing people to turn to their smaller, more secure networks.^43^ This study found that the majority of older adults maintained contact with their relatives, friends, and other acquaintances, mainly through letters, phone, email, social media, and videocall, which can be seen as essential, when personal encounters are more difficult, to maintaining social connections to reduce loneliness. This study highlights the importance of social connections in reducing loneliness in the older population and the complex relationship between loneliness, social factors, and self-rated health, which further research should address.

## Limitations and Strengths

A strength of this study is the increase in generalizability since SNAC-B is a randomized population-based sample with an even gender distribution and several well-represented age intervals. A limitation could be that the participants may have answered the question about loneliness based solely on the absence of people around them and not on perceived subjective loneliness. Also, there is a risk of attrition bias when there is a non-response in the data. In this study, non-responses were reported and handled as missing data.

## Implications for Practice

Loneliness is a complex phenomenon and can cause health problems in older adults. Understanding how to identify the older adult in loneliness requires knowledge of the manifestations of loneliness, the risk factors, and the role of social factors in loneliness. In addition, to understand how health is affected, self-reported health can be used to understand the link to perceived loneliness. All these factors are essential to understanding how to work with older adults, and guidelines should form the basis to facilitate this practice.

## Conclusion

This study showed the importance of social connections with family, friends, and others. A more extensive social network is related to less loneliness in older adults. Promoting social connections and preventing loneliness is proposed as establishing networks between younger and older generations, otherwise perceived as living parallel lives. This study highlights the importance of having someone with whom one shares a close emotional connection. There can be a strong connection with the family despite experiencing loneliness, and loneliness does not need to relate to low self-reported health. Future research could use interviews focusing on social factors and self-rated health to facilitate a deeper understanding of loneliness.

## Ethical Considerations

This study was conducted in accordance with the ethical guidelines of the Declaration of Helsinki.^44^ Participants in SNAC-B are provided written informed consent and were informed of the right to withdraw from participation at any time. Anonymity in the study was ensured. SNAC-B was approved by the ethics committee of Lund University (LU 604-00).
